# Proteomic Approach to Evaluate Mechanisms That Contribute to Food Allergenicity: Comparative 2D-DIGE Analysis of Radioallergosorbent Test Positive and Negative Patients

**DOI:** 10.1155/2011/673618

**Published:** 2011-09-19

**Authors:** Bindukumar Nair, John C. Wheeler, Donald E. Sykes, Paula Brown, Jessica L. Reynolds, Ravikumar Aalinkeel, Supriya D. Mahajan, Stanley A. Schwartz

**Affiliations:** Division of Allergy, Immunology, and Rheumatology, Department of Medicine, University at Buffalo, 640 Ellicott Street, Room 444 Innovation Center, Buffalo Niagara Medical Campus, 640 Ellicott Street, Buffalo, NY 14203, USA

## Abstract

Proteomic profiles of RAST^+^ subjects with severe food allergies and RAST^−^ subjects were compared using 2D-DIGE analysis to obtain candidate biomarkers specific to food allergies. Our analysis highlighted 52 proteins that were differentially expressed between the RAST^+^ and RAST^−^ groups of which 37 were successfully identified that include chondroitin sulfates, zinc finger proteins, C-type lectins, retinoic acid binding proteins, heat shock proteins, myosin, cytokines, mast cell expressed proteins, and MAP kinases. Biological network analysis tool Metacore revealed that most of these regulated proteins play a role in immune tolerance, hypersensitivity and modulate cytokine patterns inducing a Th2 response that typically results in IgE-mediated allergic response which has a direct or indirect biological link to food allergy. Identifying unique biomarkers associated with certain allergic phenotypes and potentially cross-reactive proteins through bioinformatics analyses will provide enormous insight into the mechanisms that underlie allergic response in patients with food allergies.

## 1. Introduction

The increased prevalence of food allergies is a significant clinical and public health problem in the USA. It is estimated that in the USA alone, food-allergic reactions accounted for over 20,000 emergency department visits, 2,300 episodes of anaphylaxis, and over 500 hospitalizations over just a 2-month period in 2003 [1, 2]. Despite the prevalence of food allergy, diagnosis and treatment are still far from optimal. Eight types of food account for over 90% of allergic reactions in affected individuals: milk, eggs, peanuts, tree nuts, fish, shellfish, soy, and wheat [3, 4]. Reactions to these foods by an allergic person can range from a tingling sensation around the mouth and lips and hives to death, depending on the severity of the allergy. Food allergy is more prevalent in children than adults. Additionally, allergic reactions to various foods often develop into allergy-related respiratory diseases such as asthma, allergic rhinitis, and atopic dermatitis. 

Several studies provide strong evidence that both host genetic susceptibility and environmental factors determine the complex regulation of IgE-mediated food allergies, however, the mechanisms by which a person develops an allergy to specific foods are largely unknown. As the IgE-mediated, “immediate type” food hypersensitivity reactions account for significant morbidity and mortality, further understanding of these mechanisms that contribute to the allergenicity could improve the prediction, diagnosis, and management of severity food allergy in individuals.

Food allergies are caused by IgE-dependent or IgE-independent immunologic reactions, which then lead to an inflammatory reaction, in which mast cells, eosinophilic granulocytes, and other cells are involved. Genetic and environmental causes predominantly underlie food allergies. Food intolerances or nonimmunologic food incompatibilities are often caused by specific enzyme deficiencies and must be diagnostically differentiated from food allergies. Constant allergen exposure and other environmental factors determine whether a sensitised individual will become chronically allergic and experience persistent symptoms. Therapeutics for the treatment of allergy are focused on prevention or diminishing the specific serum immunoglobulin E (IgE) responsible for the appearance of the allergic reaction [5, 6]. 

Proteomic technologies play an important role in drug discovery, diagnostics, and molecular medicine because they are the link between genes, proteins, and disease states. Identifying unique patterns of protein expression or biomarkers associated with IgE-mediated food allergy is a rapidly emerging area of clinical proteomics. Genomics provides only a partial picture, while proteomics identifies specific proteins responding to gene expression. Progress in protein annotation and in our understanding of protein-protein interactions will undoubtedly lead to diagnostic and therapeutic advances in the treatment of food allergies. Proteomics research technologies are rapidly changing our understanding of complex and dynamic biological systems by providing information relevant to functionally associated changes in protein abundances, protein–protein interactions, and posttranslational modifications [7–10]. 2-dimensional fluorescence difference gel electrophoresis (2D-DIGE) has emerged as a robust method to study protein expression profiling in clinical samples. 2D gel electrophoresis can simultaneously separate and display hundreds to thousands of different proteins. This method separates proteins in 2 dimensions according to their isoelectric point and their molecular size. Fluorescent, 2-D DIGE [11–13] allows the multiplex analysis of 3 sample proteomes on the same gel. Data from proteomic analysis provide enormous amount of biological information and can help establish multimodal markers for early diagnosis and prognosis. Additionally, these proteomic data can provide an opportunity for identifying molecular pathways that underlie various clinical allergic phenotypes. Identifying unique biomarkers associated with certain allergic phenotypes, and potentially cross-reactive proteins that can be identified through bioinformatics analyses will provide enormous insight into the mechanisms that underlie allergic response in patients with food allergies.

The current study examines the proteomic profile of a group of RAST^+^ subjects with severe food allergies and compares their proteomic profile with RAST^−^ subjects. Our goal is to not only identify biomarkers that are specific to food allergies that result in IgE-mediated atopy but also to identify molecular mechanism that underlie the allergic response in patients with food allergies.

## 2. Materials and Methods

### 2.1. Study Design

Both pediatric and adult patients with mild to severe food allergies who were evaluated in our allergy clinic were informed about the research study and some patients volunteered to participate. Institutional IRB approval was obtained prior to commencement of the study. Patients were consented based on the HSIRB guidelines; in case of minors, parental consent was obtained as per the IRB guidelines. A total of 10 mL of blood sample was drawn from the patient, of which a portion of the sample approx 3 mL was used for a RAST (Radioallergosorbent test) as a routine clinical procedure and the remaining sample was used for isolation of peripheral blood mononuclear cells (PBMC) which were used for the proteomics analysis. Further, gene expression studies were done using RNA which was extracted from these cells. RAST is a blood test used to detect specific IgE antibodies to suspected or known allergens, in this case, specific to food allergens. The IgE antibody is associated with Type I allergic response. Based on the results of the RAST test, the patients were classified into RAST positive (RAST^+^) or RAST negative (RAST^−^). A total of *n* = 9 RAST^−^ and *n* = 12 RAST^+^ patients were recruited for the study. Table 1 provides demographic and clinical information for the food RAST^+^ and RAST^−^ subjects. Comparative analysis was done between RAST^+^ and RAST^−^ patient groups using 2D-DIGE proteomic analysis. Three independent sample pools from RAST^+^ versus RAST^−^ patient samples were analyzed by 2D-DIGE for identifying the differentially expressed proteins. Each pooled sample consisted of 3 RAST^+^ and 3 RAST^−^patient samples, respectively.

### 2.2. PBMC Isolation

Peripheral blood leukocytes were isolated from whole blood using Ficoll-Hpaque gradient (Amersham-Pharmacia, Piscataway, NJ, Cat number 17-1440-03). Samples were centrifuged for 30 min at 700 ×g and 20°C without applying a brake. The PBMC interface was carefully removed by pipetting and was washed twice with PBS/EDTA and resuspended in 2 mL of complete RPMI media and total number of cells counted using a hemocytometer.

### 2.3. RNA Extraction

Cytoplasmic RNA is extracted using Trizol reagent (Invitrogen- Life Technologies, Carlsbad, Calif) [14]. The amount of RNA is quantitated using a Nanodrop ND-1000 spectrophotometer (Nanodrop Wilmington, Del) and isolated RNA is stored at −80°C until used.

### 2.4. Real Time Quantitative PCR (Q-PCR) 

Q-PCR was used to quantitate gene expression. RNA is reverse transcribed to cDNA using the reverse transcriptase kit from Promega (Promega Inc, Madison, Wis, USA; Cat number A3500). Relative abundance of each mRNA species is quantitated by real time quantitative PCR for specific primers using the Brilliant SYBR green QPCR master mix from Stratagene (Stratagene Inc, La Jolla, Calif, USA; Cat number 600548-51). Relative expression of mRNA species is calculated using the comparative C_T_ method [15]. All data are controlled for quantity of RNA input by performing measurements on an endogenous reference gene, *β*-actin. In addition, results on expression levels of different genes from Rast^+^ samples are normalized to results obtained from Rast^−^ samples. Results are expressed as transcript accumulation index (TAI) as described earlier [16]. This calculation assumes that all PCR reactions are working with 100% efficiency.

### 2.5. Protein Extraction

 PBMC isolated from RAST^+^ and RAST^−^ subjects were washed twice with 1X PBS (Invitrogen, Grand Island, NY, USA). Total protein was extracted using standard cell lysis buffer (30 mM TrisCl; 8 M urea; 4% (w/v) CHAPS, adjusted to pH 8.5) for 10 min on ice. The cell lysate was centrifuged at 4°C for 10 min at 12000 ×g and was further purified by precipitation with chloroform/methanol as described [17]. Samples were resuspended in standard cell lysis buffer. Protein concentrations were determined using the Coomassie Protein Reagent (Bio-Rad, Hercules, Calif, USA) prior to DIGE analysis.

### 2.6. Two-Dimensional Difference Gel Electrophoresis (2D-DIGE)

The Ettan DIGE technique developed by GE Healthcare (Piscataway, NJ, USA) was used to detect differences in protein abundance between the RAST^+^ and RAST^−^ samples. The Ettan DIGE system uses 3 CyDye DIGE fluors (Cy2, Cy3, Cy5), each with a unique fluorescent wavelength, matched for mass and charge. CyDyes form a covalent bond with the free epsilon amino group on lysine residues of the sample proteins. CyDyes label approximately 2% of the lysine residues. This system allows in each set of gel 2 experimental samples and an internal standard to be simultaneously separated on the same gel. The internal standard is comprised a pool of an equal amount of all the experimental samples. The use of an internal standard facilitates accurate inter-gel matching of spots, and allows for data normalization between gels to minimize gel to gel experimental variability [12]. Cell lysates were labeled with CyDye per the manufacturer. All reagents used were from GE Healthcare (Amersham Biosciences, Piscataway, NJ, USA). 

Three independent (*n* = 3) 2D-DIGE experiments were done comparing RAST^+^ and RAST^−^ patient samples. Briefly, six samples ( 3 RAST^+^ and 3 RAST^−^), each of 50 *μ*g cell lysate were labeled with 400 pmoL of either Cy3 or Cy5, kept on ice for 30 min and then quenched with a 50-fold molar excess of free lysine. 50 *μ*g of protein pooled from equal amounts of protein from all the six experimental samples were labeled with Cy2 and used as an internal standard in each of the three sets of Cy3/Cy5 gels. Cy3-, Cy5-, and Cy2-labeled samples and unlabelled protein (500–1000 *μ*g) were pooled. Unlabeled protein was added to enhance the protein staining and hence a better image quality of the SYPRO ruby staining. An equal volume of 2X sample buffer (8 M urea; 2% (v/v) Pharmalytes 3–10; 2% (w/v) dithiothreitol (DTT); 4% (w/v) CHAPS) was added and incubated on ice for 10 min. The total volume of sample was adjusted to 450 *μ*L with rehydration buffer (4% (w/v) CHAPS; 8 M urea; 1% (v/v) Pharmalytes 3–10 nonlinear (NL); 13 mM DTT). Samples were applied to immobilized pH gradient (IPG) strips (24 cm, pH 3–10 NL) and absorbed by active rehydration at 30 V for 13 hr. Isoelectric focusing was carried out using an IPGphor IEF system with a 3-phase program; first phase at 500 V for 1 hr, second phase at 1000 V for 1 hr, and third phase (linear gradient) 8000 V to 64000 V for 2 hr (50 uA maximum per strip). Prior to separation in the second dimension, strips were equilibrated for 15 min in equilibration buffer I (50 mM Tris-HCl, 6 M urea, 30% (v/v) glycerol, 2% (w/v) SDS, 0.5% (w/v) DTT). The strips were again equilibrated for 15 min in equilibration buffer II (50 mM Tris-HCl, 6 M urea, 30% (v/v) glycerol, 2% (w/v) SDS, 4.5% (w/v) iodoacetamide) and the equilibrated IPG strips were transferred onto 18 × 20 cm, 12.5% uniform polyacrylamide gels poured between low fluorescence glass plates. Gels were bonded to inner plates using Bind-Silane solution (Promega, Madison, Wis, USA) according to the manufacturer. Strips were overlaid with 0.9% agarose in 1X running buffer containing bromophenol blue and were run for 16 hr (1.8 W/gel overnight) at 15°C in an Ettan DALT electrophoresis system. After the run was completed, the 2D gels were scanned 3 times with a Typhoon 9410 imager, each time at different excitation wavelengths (Cy3, 532 nm; Cy5, 633 nm; Cy2, 488 nm). Images were cropped with ImageQuant v5.2 software and then imported into DeCyder differential in-gel analysis (DIA) v5.0 software from GE Healthcare for spot identification and normalization of spot intensities within each gel. 

 Gels were fixed in 30% (v/v) methanol, 7.5% (v/v) acetic acid for 3 hr and stained with SYPRO-ruby dye (Molecular Probes, Eugene, Ore, USA) overnight at room temperature. Gels were destained in water and then scanned using the Typhoon 9410 scanner. Spots of interest were excised from the gel using the Ettan Spot Picker. DeCyder software (GE Healthcare) was specifically developed for use with the Ettan DIGE system. DeCyder software allows for automatic detection of spots, background subtraction, quantitation, normalization, internal standardization, and integral matching. The differential in-gel analysis (DIA) component of DeCyder software draws boundaries around spots in a composite gel image obtained from the intragel overlap of the Cy2-, Cy3-, and Cy5- scanned images and normalizes the data from each CyDye to account for differences in dye fluorescence intensity and scanner sensitivity. For all the three sets of experiments (six images), the abundance difference between samples (RAST^+^ and RAST^−^) run on the same gel was then analyzed. The biological variation analysis (BVA) component of DeCyder software was then used to match all image comparisons from individual Cy3/Cy5 gel sets for a cross-gel statistical analysis. DeCyder BVA initially calculates normalized intensities (standard abundance) for all spots by comparison to the internal standard, and from this, an average volume ratio and a Student's paired *t*-test derived *P* value were calculated for each spot. A paired *t*-test derived *P* value of ≤0.05 was considered statistically significant [12].

### 2.7. Mass Spectrometry and Protein Identification

Spots were excised from 2D-gels loaded with 500 to 1000 *μ*g of total protein and stained with Sypro ruby (Molecular Probes). The gel plugs were incubated in 200 *μ*L of 200 mM ammonium bicarbonate with 50% acetonitrile for 30 min at 37°C and then dried with a Speed Vac. Samples were then incubated for 1 h at room temperature in 3.5 *μ*L of 20 *μ*g/mL trypsin (Promega) in 40 mM ammonium bicarbonate. An additional 30 *μ*L of 40 mM ammonium bicarbonate with 10% acetonitrile was added and samples were incubated overnight at 37°C. The samples were desalted using *μ* C_18_ ZipTips (Millipore, Bedford, Mass, USA) and eluted directly onto a MTP ground steel 384-target (Bruker Daltonics, Billerica, Mass, USA) with the matrix solution (a saturated solution of *α*-cyano-4-hydroxy-cinnamic acid [CHCA: Bruker Daltonics] dissolved in 0.1% trifluoroacetic acid:75% acetonitrile). MALDI spectra in positive ion mode were obtained using a Bruker Daltonics Biflex IV MALDI-TOF mass spectrometer operating in the reflectron mode. The spectra in the range of 500–3200 Da were obtained by the summation of 50–200 laser shots. Identification and labeling of the peaks were done manually. External calibration was carried out using the peptide calibration standard II (700–4000 Da) (Bruker Daltonics, number 222570). Data acquisition was executed using the Flex Control program (Build 2.4.0.0) and data processing was performed using the Bruker XTOF 5 software (version 5.1.5). The signatures of proteins of interest formed by the masses of the products of tryptic digestion were analyzed using an on-line search engine MASCOT (http://www.matrixscience.com/) to compare the calculated masses of a theoretical tryptic digestion. Searches were conducted for matching masses from theoretical tryptic peptides from the NCBI protein databases. We used the following search parameters: monoisotopic masses, with a tolerance of 200 ppm or less (typically 75–100 ppm or 0.1–0.5 Da), one missed tryptic cleavage, a carbamidomethyl modification of cysteine (to account for iodoacetamide treatment) and variable modifications of oxidation of methionine and phosphorylation of serine, threonine or tyrosine. A protein identification was considered significant for a Mowse score with *P* < 0.05.

## 3. Results

### 3.1. 2D-DIGE Analysis and Protein Identification

Protein profiles of RAST^−^ and RAST^+^ PBMCs were analyzed by DIGE to examine the differential expression of proteins among the two groups. Protein samples from RAST^+^ and RAST^−^ PBMC cell lysates were labeled with fluorescent CyDyes, pooled and analyzed in triplicate by 2D-DIGE. The pooled mixture of equal amounts of protein from each of the six experimental samples labeled with Cy2 and run on every gel provided an internal standard to ensure that every protein in each sample appears on all of the gels. Each Cy3- or Cy5-labeled sample was then compared to the same internal standard and measurements were taken relative to the internal Cy2 standard, thereby reducing gel-to-gel variation and increasing statistical confidence. Dye-swapping was performed to minimize labeling-dependent bias. 

**Figure 1:**
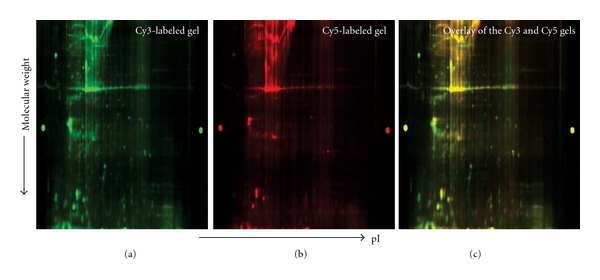
Representative 2D-DIGE gel images of Cy3 and Cy5 Dye-labeled RAST^+^ and RAST^−^ samples. Protein samples were labeled with Cy Dyes and analyzed by 2D-DIGE. (a) RAST^−^ (Cy3); (b) RAST ^+^ (Cy5); (c) overlay of Cy3 and Cy5 gel images.

**Figure 2:**
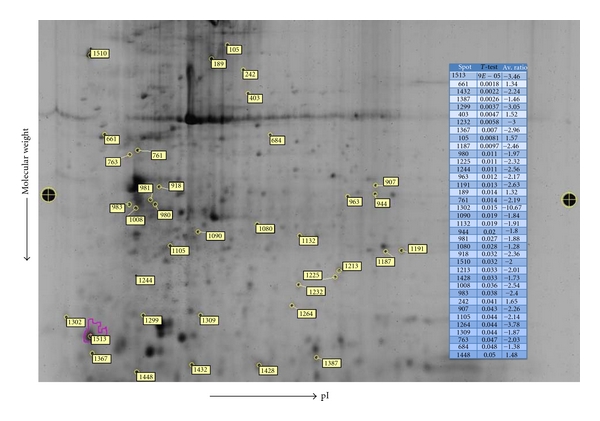
SYPRO ruby-stained 2-D gel image of a 2D-DIGE separation of RAST^+^ and RAST^−^ samples. 2-D electrophoresis was performed using 24 cm, pH 3–10 linear IPG strips and 10% SDS-PAGE gels. Spots that showed statistically significant differences in intensity between the RAST^−^ and RAST^+^ are marked. Differences in Cye Dye-labeled sample abundances were analyzed using DeCyder Software. Differentially expressed protein spots with their respective fold differences and *P* values are given as a table. A paired *t*-test derived *P* value of ≤0.05 was considered statistically significant. Protein spots were excised and identified using MALDI-TOF and the identified proteins are listed in Table 2.

The DIA (differential in-gel analysis) module of the differential analysis software DeCyder (GE Healthcare) was used to calculate protein spot volumes and the normalized volume ratio for each differentially labeled comigrated protein. On an average of about 1000 spots were detected and matched successfully in three RAST^+^ and RAST^−^ CyDye-labeled proteome gels (Figure 1). The ratio of spot intensity of the two groups of protein spots is then calculated. Gel to gel matching of the standard spot maps from each gel was performed using the DeCyder BVA software module. This allowed for the statistical analysis of changes in protein abundance between samples. Statistical analysis (paired *t*-test) was performed for the difference between the abundance of proteins from RAST^+^ cells versus proteins from and RAST^−^ cells. A total of 52 protein spots showed a significant change in abundance with *P* < 0.05. Proteins from 37 gel spots were successfully identified. Figure 2 is the SYPRO ruby-stained 2D gel image showing all protein spots. Differentially expressed protein spots showing significant modulation were selected for subsequent identification and are shown as numbered outlines. The maximum increase in protein expression was 1.65-fold (spot number 1302 in Figure 2) and the maximum decrease observed was 10.7-fold (spot #242, Figure 2).

### 3.2. MALDI_TOF Analysis for Protein Identification

Proteomic evaluation of differential expression of proteins in PBMCs of RAST^+^ and RAST^−^ subjects by 2D-DIGE and MALDI-TOF resulted in a short list of 37 proteins that were found to be significantly modulated (Table 2). The table lists these proteins and includes their symbols, % sequence coverage of the identified peptide, number of matching peptides, theoretical mass, and calculated pI of the identified protein.

### 3.3. Validation of the Differentially Expressed Proteins

**Figure 3:**
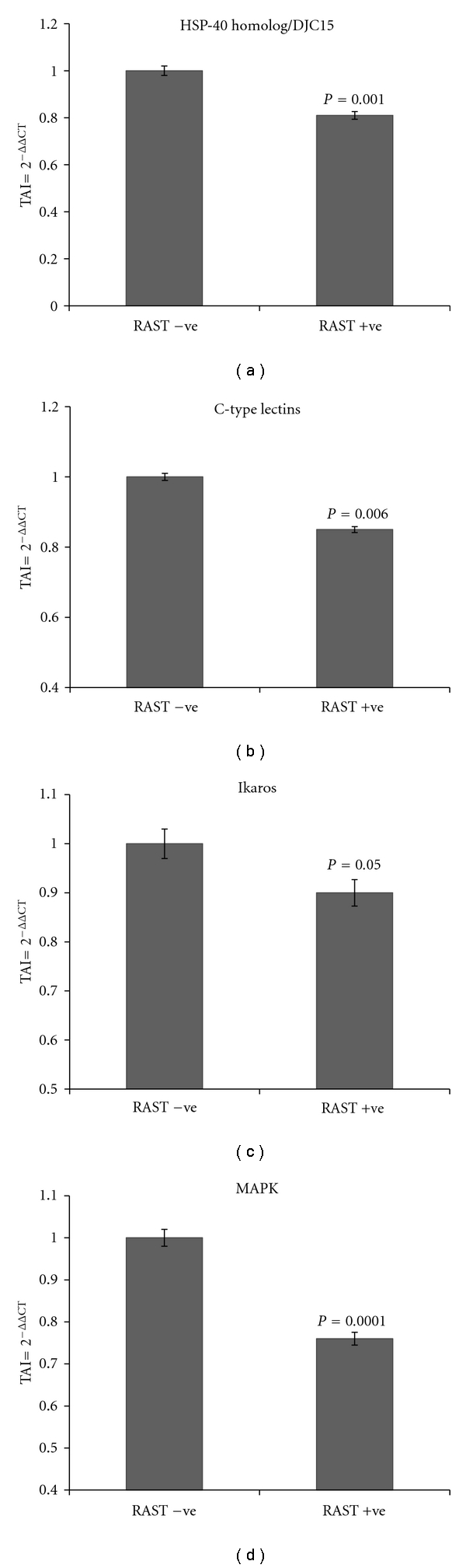
Relative gene expression of heat shock protein-40 homolog (DJC15); C-type lectins, Ikaros; MAPK in RAST^+^ versus RAST^−^ Patients as quantitated by QPCR. Gene expression analysis of heat shock protein-40 homolog; C-type lectins, Ikaros; MAPK were done using RNA extracted from PBMC from RAST^+^ and RAST^−^ subjects. RNA was reverse transcribed and mRNA expression levels of the above genes were quantitated using real time QPCR. Relative expression of mRNA species was calculated using the comparative C_T_ method. Data are the mean ± SD of 3 separate experiments done in duplicate. Data are presented as TAI or the transcript accumulation index. Statistical significance was determined by Student's *t*-test based on comparison between the RAST^+^ versus RAST^−^ patients.

**Figure 4:**
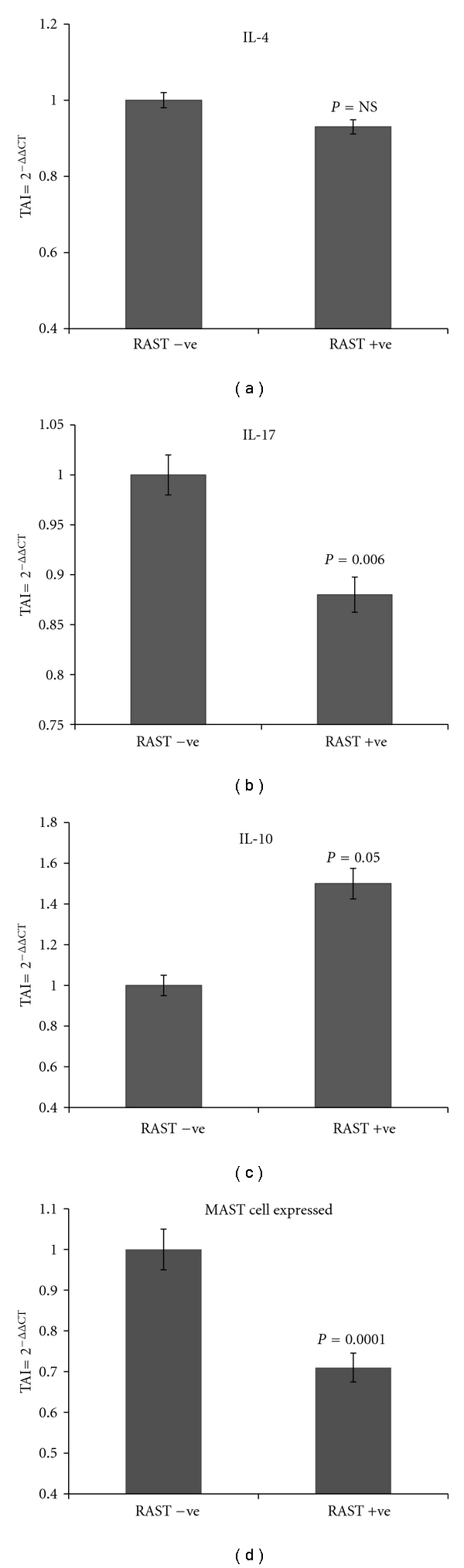
Relative gene expression of retinoic acid-binding protein; chondroitin transferase; myosin; zinc finger protein; in RAST^+^ versus RAST^−^patients as quantitated by QPCR. Gene expression analysis of retinoic acid-binding protein; chondroitin transferase; myosin; zinc finger protein; was done in RNA extracted from PBMC from RAST^+^ and RAST^−^ subjects. RNA was reverse transcribed and mRNA expression levels of the above genes was quantitated using real time QPCR. Relative expression of mRNA species was calculated using the comparative C_T_ method. Data are the mean ± SD of 3 separate experiments done in duplicate. Data are presented as TAI or the transcript accumulation index. Statistical significance was determined by Student's *t*-test based on comparison between the RAST^+^ and RAST^−^ patients.

**Figure 5:**
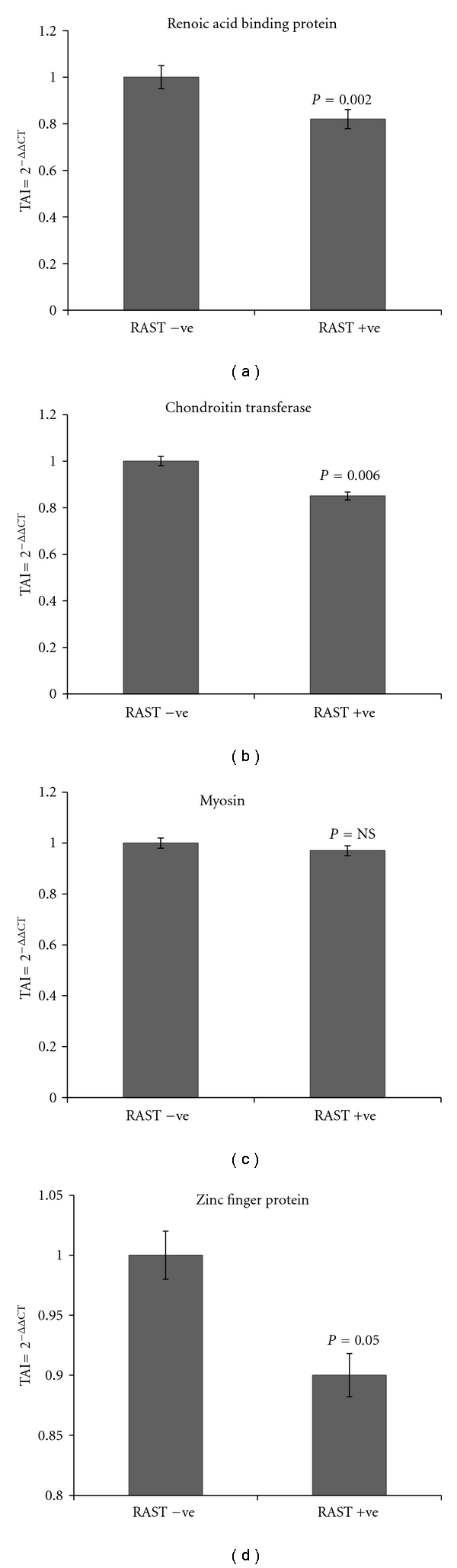
Relative gene expressions of IL-4, IL-17, IL-10, and MCEM1 in RAST^+^ versus RAST^−^ patients as quantitated by QPCR. Gene expression analysis of IL-4, IL-17, IL-10, and MCEM1 was done in RNA extracted from PBMC from RAST^+^ and RAST^−^ subjects. RNA was reverse transcribed and mRNA expression levels of the above genes were quantitated using real time QPCR. Relative expression of mRNA species was calculated using the comparative C_T_ or the ΔΔCt method. This method calculates fold changes between each normal sample and its paired affected sample. ΔCt represents normalized gene expression level (normalized to its own internal control housekeeping gene *β*-actin). ΔCt for test and control samples were averaged and compared to obtain the ΔΔCt value (an average value). CV, or coefficient of variation, is calculated using the formula STDEV/average. Data are the mean ± SD of 3 separate experiments done in duplicate. Data are presented as TAI or the transcript accumulation index. Statistical significance was determined by Student's *t*-test based on comparison between the RAST^+^ versus RAST^−^ patients.

Having shortlisted these key proteins that are differentially expressed in the RAST^+^ and RAST^−^ subjects, we evaluated if the gene expression levels of these proteins were significantly different in these two groups. Based on an exhaustive literature review and on comparative analysis using gene expression studies by quantitative PCR, we have highlighted a group of key proteins that play a role in food allergenicity. These include chondroitin sulfates, zinc finger proteins, C-type lectins, retinoic acid-binding proteins, heat shock proteins, membrane proteins such as myosin and cytokines such as IL-17F, certain mast cell expressed proteins, and signal transduction molecules such as the family of MAP kinases. Almost all of these proteins play a role in immune tolerance and hypersensitivity [18–22], and they modulate cytokine patterns that favor a Th2 response that typically results in IgE-mediated allergic response. The following are the gene expression levels of the key proteins that were significantly lower in RAST^+^ subjects as compared to RAST^−^ subjects. Heat shock protein-40 homolog (DJC15) (19% decrease, *P* = 0.001); C-type lectins (15% decrease; *P* = 0.006); MAPK (24% decrease; *P* = 0.0001); retinoic acid-binding protein (18% decrease; *P* = 0.002); chondroitin transferase (15% decrease; *P* = 0.006); zinc finger protein (10% decrease; *P* = 0.05); IL-17F protein (12% decrease; *P* = 0.02); and MCEM1 mast cell expressed membrane protein 1 (29% decrease; *P* = 0.0001). The gene expression levels of cytokine IL-10 were significantly higher (50% increase; *P* = 0.0001) in the RAST^+^ subjects as compared to the RAST^−^ subjects. No significant differences in the gene expression levels of myosin and Ikaros were observed between the 2 patient groups. Figures 3, 4, and 5 show the fold regulation in the gene expression levels of these key proteins. Although the fold changes in gene and protein changes in these key molecules are not of similar magnitude, the trends in their expression are in concordance and therefore suggests that these proteins may play a significant role in the development of food allergies.

### 3.4. Biological Network Analysis Using MetaCore

**Figure 6:**
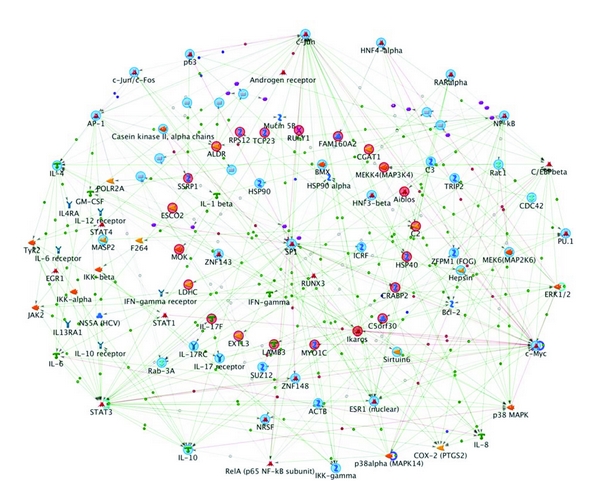
The biochemical processes networks of the differentially expressed proteins generated by MetaCore software using a transcription regulation algorithm. The biochemical processes regulation networks were ranked by a *P* value and interpreted in terms of GO. Gene network illustrates proteins and interactions of the differentially expressed proteins in RAST^+^ and RAST^−^ patients. The network was obtained using analyze network algorithm of MetaCore and illustrates the close clustering and interconnectedness of biochemical process networks of the identified differentially regulated proteins in food allergy. Colored highlighted symbols (nodes) represent proteins (enzymes (yellow arrows), transporters (purple shapes), receptor ligands (green shapes), transfactors (red shapes), and other proteins (blue shapes)). Red solid circles correspond to differentially expressed proteins in RAST^+^ samples identified by MALDI analysis. Blue circles show the proteins in the network within the immediate vicinity of those identified proteins. The small colored hexagons on vectors between nodes describe positive interaction (green), unspecified interactions (black), or logical relationships (blue). Each connection (vector) represents a direct interaction between proteins.

Given the high through put data that emerges from proteomic analysis, additional data analysis using novel software tools, can be used to interrogate the biological context of lists of genes and proteins. We used one such software tool, MetaCore, which uses a proprietary, manually curated database of human protein-protein, protein-DNA, and protein compound interactions, metabolic and signaling pathways which is supported by proprietary ontologies. The transcription regulation networks of differentially expressed proteins in the RAST^+^ and RAST^−^ groups were generated by MetaCore software using a transcription regulation algorithm. The networks were ranked by a *P* value and interpreted in terms of GO. The list of significantly regulated proteins emerged after 2D-DIGE/MALDI-TOF analysis of the RAST^+^ and RAST^−^ samples, highlighted in Table 2, was entered in to MetaCore. The MetaCore software has in-built algorithms that create biological networks from the uploaded protein list and assign biological processes to the generated networks. We built these various networks using “Analyze Network” algorithm of MetaCore which is useful in providing additional information that is not present in the original list of root nodes. 37 differentially expressed proteins identified on comparison between the RAST^+^ and RAST^−^ groups were mapped into the software and subsequently analyzed. Networks were generated as per *P*  value, Z-score and G-score. *P* value is based on the saturation of each sub-network with proteins from the user selected list. Z-score ranks the subnetworks according to saturation with the objects from the initial list of seed objects. The Z-score ranks the sub-networks of the analyze network algorithm with regard to their saturation with proteins from the experiment. The G-score modifies the Z-score based on the number of canonical pathways used to build the network. If a network has a high G-score, it is saturated with expressed proteins (from Z-score) and it contains many canonical pathways. We chose the top 3 networks from the search results as ranked by both G-score and *P* values. The significance of association between the biological processes and the differentially expressed proteins were represented by *P* values. The list of most significant GO processes associated with the network is shown in Table 3 along with the respective *P* values. The top 3 processes, the percentage of target nodes involved and the corresponding *P* values are positive regulation of immune system process (35%, *P*-value 2.2*e* − 50), MyD88-independent toll-like receptor signaling pathway (19.32%, 2.51*e* − 49) and toll-like receptor 3 signaling pathway (19.32%, 4.1*e* − 49).  Figure 6 illustrates the close clustering and interconnectedness of biochemical process networks of the identified proteins which are differentially regulated.

## 4. Discussion

Allergic responses to food antigens involve a state of immediate hypersensitivity to certain food proteins. The mechanism underlying the initiation and development of allergic responses involves cytokine activation which is believed to directly induce the differentiation of effector Th2 lymphocytes; these Th2 responses play a pivotal role in the development of allergic responses to specific food antigenic triggers. In this paper, we utilized a high throughput proteomic approach, 2D-DIGE, coupled with MALDI-TOF protein identification methods to study the differential expression of proteins in two groups of RAST^+^ and RAST^−^ subjects with the goal to identify key proteins that may play a role in the development of food allergenicity. In contrast to traditional biochemical approaches that can only study one or a few particular protein species, proteomics analysis allows the simultaneous analysis of thousands of proteins. This is the first comprehensive differential proteomic analysis of PBMC obtained from RAST^+^ and RAST^−^ subjects. Over 1,000 distinct proteins were identified of which about 52 proteins displayed significant differential expression between the RAST^+^ and RAST^−^ groups. Of these 52 significantly regulated proteins, 37 were successfully identified (Table 2). A large proportion of these proteins have direct or indirect biological relevance to food allergy. 

Food allergenicity is characterized by abnormal IgE production, peripheral eosinophilia, mast cell activation, and induction of Th2 lymphocytes expressing cytokines such as IL-4 and IL-10 [23, 24]. Even though we could not observe any differential expression of IL-4 and IL-10 in the 2D-DIGE analysis, Metacore analysis indicated a very active involvement of these molecules in the networks. A further QPCR analysis showed a significant increase in IL-10 levels in RAST^+^ patients as compared to RAST^−^ patients. IL-10 is a cytokine that downregulates both Th1 and Th2 cytokine production [25], and the fact is that its levels are significantly elevated in RAST^+^ patients and may be useful in identifying human clinical tolerance to foods. IL-10 is also believed to help differentiation of T helper cells into the Th2 phenotype, thereby contributing to the development of allergic responses to food antigens. IL-4 levels in RAST^+^ patients were lower but not statistically significant as compared to RAST^−^ patients. A decrease in IL-4 is believed to impair the development of food allergy and the aversion to antigen. Production of IL-4 and levels of specific IgE/IgG1 antibodies correlate with aversion to antigen induced by food allergy in mice [26]. The mechanism underlying the initiation and development of allergic responses involves IL-4 that directly induces the differentiation of committed effector Th2 lymphocytes, and Th2 responses play a pivotal role in the development of allergic responses. IL-4 inhibits the induction of Foxp3 and the generation of inducible regulatory T cells and may also function as anti-inflammatory cytokine [27]. 

IL 17F is reported to be involved in inflammation and a study on effect of IL-17/IL-17F deficiency in an allergic asthma IL-17 KO mice model, exhibited reduced Th2 cytokine expression, whereas IL-17F KO mice showed elevated type 2 cytokines and eosinophil functions compared with WT mice. This indicates that IL-17 and IL-17F may have opposite functions in chronic allergic airway diseases [28]. The precise role of IL-17 and a new population of IL-17-producing Th cells (Th17) in allergic inflammation is unclear, but Th17 cells are characterized by the production of inflammatory cytokines such as IL-17A, IL-17F, IL-22, and IL-26 [29]. The highest percentage of IL-17-producing cells has been found in severe atopic dermatitis, suggesting a direct correlation between the presence of Th17 cells and severity of the disease [30], indicating that there is possible cross-talk between Th17 cells and eosinophils. Our observation of a significant decrease in IL-17 in RAST^+^ patients is contradictory to what we expected based on its role as a promoter of allergic inflammation. This observation leads us to believe that IL-17 may be associated with patterns of cytokine dysregulation that are associated with specific atopic phenotypes, though the precise mechanism of its modulation is unclear. 

 Mast cells are believed to be the key effector cells of IgE-mediated anaphylactic reaction, therefore, a modulation in the expression of the mast cell expressed protein MCEM1 in RAST^+^ patients is anticipated. We observed a significant decrease in MCEM1 expression in RAST^+^ patients. The MAPK pathway and its downstream effectors play an important role in the regulation of cytokine expression in mast cells. Signals emanating from many cell surface antigenic receptors and environmental stimuli can converge on the MAPK pathway, which in turn phosphorylates and activates various transcriptional factors and molecular effectors, thereby modulating allergic response. Proteins such as zinc finger proteins, retinoic acid-binding proteins, chondroitin transferase, C-type lectins and heat shock proteins in the soluble microenvironment are believed to be involved in the conditioning of antigen presenting cells, thereby promoting tolerance to dietary antigen primarily via the induction of regulatory T cells. 

Yet another unique protein that is significantly modulated in RAST^+^ patients is Ikaros. This family of proteins plays an important role in hematopoietic development and is believed to modulate the levels of an immunosuppressive compound called bis(tributyltin)oxide (TBTO) that is present in the diet and which can contribute significantly to the development of food allergy to peanut or ovalbumin. TBTO can decrease allergen-specific Th2 cytokine production, number of eosinophilic and basophilic granulocytes in the blood and production of mast cell proteases after an oral food challenge. 

To understand the relevance of these proteins and their role in allergy or overall inflammation and to ascertain their interaction with other proteins in known networks the identified proteins were analyzed using Metacore network building tools. Of the total 37 identified proteins imported to Metacore, 20 were successfully mapped in the networks (Figure 6). The unconnected proteins in the network were removed. The proteins differentially regulated in RAST^+^ subjects were shown in red circles in the network. In protein network generated using the uploaded, differentially expressed proteins in this study showed significant links to inflammatory and immunological responses. Among the upregulated proteins all except Globin-1 could be mapped on to the network.

## 5. Conclusion

The current investigation has helped to identify key proteins involved in food allergenicity that provide evidence for a relationship between Th2 immune response and food allergy and suggests that these responses play an important role in the development of food allergy. This study provides important information about global protein expression in RAST^+^ patients with food allergies. Although we could not subdivide the patients with food allergies into specific food type, namely, peanut, egg, shell fish, and so forth, the identification of these key proteins provides clues to elucidate mechanisms of the structures that contribute to allergenicity, which thus, in turn, would help alleviate food allergens. Further, these key proteins can be utilized as diagnostic markers that will not only help in the diagnosis and management of food allergy but also may be used in future immunotherapy.

## Figures and Tables

**Table:** (a) Food rast negative patients.

Pt number	Age	Gender	Ethnicity	Clinical H/O	Skin test	RAST Antigen-specific IgE (ng/mL)
1	3	F	White	Peanut: urticaria and rash after peanut food challenge	Negative: peanut 2/4*	Negative: peanut and tree nuts
2	15	F	Black	Tree nut: anaphylaxis (angioedema, shortness of breath and abdominal pain)	Not done	Negative: peanut and tree nuts
3	1	M	White	Eczema workup	Positive: milk 12/20*	Negative: egg, milk and wheat
4	2	M	Hispanic	Eczema workup	Negative: egg 5/6*	Negative: egg
5	3	M	Black	Fish: urticaria	Positive: mixed Whitefish	Negative: tuna, salmon, cod
6	8	F	White	Cocoa: headache, strawberry: rash	Positive: cocoa 5/45* Negative: fresh strawberry	Negative: cocal and strawberry
7	7	M	Not specified	Peanut: hives, egg: hives	Not done	Negative: egg, peanut and tree nuts
8	7	F	White	Tuna: hives	Negative: tuna 4/5* (negative)	Negative: tuna
9	9	M	Not specified	Tomato: facial edema	Not done	Negative: tomato

**Table:** (b) Food rast positive patients.

Pt number	Age	Gender	Ethnicity	Clinical H/O	Skin test	RAST Antigen-specific IgE (ng/mL)
1	6	M	Black	Peanut: angioedema, shortness of breath and itchy eyes	Not done	Peanut: >100
2	4	M	Black	Peanut: rash	Positive: peanut (5/20)*	Peanut: 0.86
3	5	F	Black	Peanut: hives, soy: eczema flair	Positive: soy (5/10) and egg (3/7)	Peanut: 7.32, soy: 5.15 and tree nuts: 4.14–>100
4	3	M	Black	Peanut: hives	Positive: peanut (12/20)*	Peanut: 72.8
5	2	F	White	Peanut: eczema flare	Positive: peanut (15/40)*	Peanut: 4.07
6	6	M	White	Tree nut: difficulty breathing	Not done	Tree nuts: 15–>100
7	16	M	Black	Crab: angioedema	Positive: crab (10/12)*, lobster (10/13)* and clam (4/6)*	Crab: 14.70, Clam: 1.06 and lobster: 9
8	4	M	Not specified	Peanut: anaphylaxis	Not done	Peanut: 9.39
9	11	M	Black	Cashew: throat itching	Positive: cashew (9/9)* and walnut (18/20)*	Tree nuts: 0.51–9.24
10	16	F	Black	Peanut: itchy throat	Not done	Peanut: 20
11	1.5	F	Black	Egg: anaphylaxis	Not done	Egg: 62.80
12	9	F	White	Egg: urticaria	Negative	Egg: 2.78

**Table 2:** List of the up/down regulated proteins from RAST^+^ and RAST^−^ samples analyzed by 2D-DIGE and further identified by MALDI-TOF. Data shown in the table include spot number, calculated mass, theoretical pI, number of matching peptides, % sequence coverage, name and symbol of identified proteins.

Spot number	Mass	*P*I value	Peptides matches	Sequence coverage (%)	Protein name	Symbol
189	14505	6.81	7	59	40S ribosomal protein S12	RS12_HUMAN
1244	54005	6.21	11	28	6-phosphofructo-2-kinase/fructose-2,6-biphosphatase 4	F264_HUMAN
1008	35830	6.51	7	32	Aldose reductase	ALDR_HUMAN
1213	15683	5.42	6	27	Cellular retinoic acid-binding protein 2	RABP2_HUMAN
1302	61312	8.63	8	17	Chondroitin sulfate N-acetylgalactosaminyltransferase 1	CGAT1_HUMAN
1387	91751	5.98	15	19	Coiled-coil domain-containing protein	CF204_HUMAN
1299	83214	7.23	8	17	Complement C2	CO2_HUMAN
1428	36702	8.45	10	39	C-type lectin domain family 4 member K	CLC4K_HUMAN
963	38901	5.27	9	26	DDB1- and CUL4-associated factor 7	DCAF7_HUMAN
1105	40517	9.00	10	28	Developmentally regulated GTP-binding protein 1	DRG1_HUMAN
1225	30836	8.71	6	18	DnaJ homolog subfamily C member 27	DJC27_HUMAN
416	16373	10.08	7	59	DnaJ(HSP40) homolog	DJC15_HUMAN
944	77221	5.36	11	21	Elongation factor G 2	EFG2_RALME
1448	104682	6.08	12	12	Exostosin-like 3	EXTL3_HUMAN
105	81024	6.45	13	20	FACT complex subunit SSRP1	SSRP1_HUMAN
661	16459	8.67	6	51	Globin-1 OS=Petromyzon marinus PE=1 SV=2	GLB1_PETMA
1309	37162	10.80	8	31	Heparan sulfate glucosamine 3-O-sulfotransferase6	HS3S6_HUMAN
1080	57528	6.12	14	22	IKAROS zinc finger protein 1	IKZF1_HUMAN
1513	18033	9.15	6	49	Interleukin-17F	IL17F_HUMAN
980	129489	7.14	11	12	Laminin subunit beta-3	LAMB3_HUMAN
403	36288	7.08	8	24	L-lactate dehydrogenase C chain	LDHC_HUMAN
242	47983	9.64	9	23	MAPK/MAK/MRK overlapping kinase	MOK_HUMAN
1132	21215	9.03	7	24	Mast cell expressed membrane protein 1	MCEM1_HUMAN
684	181570	5.94	13	17	Mitogen-activated protein kinase kinase kinase 4	M3K4_HUMAN
907	121648	9.46	11	14	Myosin-Ic	MYO1C_HUMAN
1191	60539	9.62	19	28	Parafibromin	CDC73_HUMAN
1187	43939	9.33	10	24	Pentatricopeptide repeat-containing protein 2	PTCD2_HUMAN
1090	56852	8.52	10	21	Probable histidyl-tRNA synthetase, mitochondrial	SYHM_HUMAN
761	50550	5.00	10	34	Rab GDP dissociation inhibitor alpha	GDIA_HUMAN
983	79767	5.54	13	18	RUN and FYVE domain-containing protein 1	RUFY1_HUMAN
981	12439	10.54	7	73	Somatoliberin	SLIB_HUMAN
763	33047	6.64	7	31	Tissue factor	TF_HUMAN
918	23294	11.33	7	44	Transcription factor 23	TCF23_HUMAN
1264	64218	8.56	9	23	Transmembrane anterior posterior transformation protein 1 homolog	TAPT1_HUMAN
1510	121711	5.95	16	22	Transmembrane protein 132C OS	T132C_HUMAN
1432	23069	9.51	6	38	UPF0684 protein C5orf30	CE030_HUMAN
1367	57986	6.11	8	19	Zinc finger protein Aiolos	IKZF3_HUMAN

**Table 3:** List of the most significant gene ontology (GO) processes associated with the biological network shown in Figure 6 as determined by MetaCore analysis along with the corresponding *P* values which indicates that the key protein identified by proteomic analysis are associated with the listed biological processes in a prioritized manner.

Number	Biological process	% involvement in the biological process	*P* value
1	Positive regulation of immune system process	35.23	2.280 *e* − 50
2	MyD88-independent toll-like receptor signaling pathway	19.32	2.516 *e* − 49
3	Toll-like receptor 3 signaling pathway	19.32	4.103 *e* − 49
4	Toll-like receptor signaling pathway	19.89	5.703 *e* − 48
5	Signal transmission via phosphorylation event	32.95	8.113 *e* − 48
6	Intracellular protein kinase cascade	32.95	8.113 *e* − 48
7	Positive regulation of response to stimulus	33.52	3.663 *e* − 47
8	Pattern recognition receptor signaling pathway	19.89	8.743 *e* − 47
9	Activation of innate immune response	19.89	2.639 *e* − 46
10	Innate immune response-activating signal transduction	19.89	2.639 *e* − 46
11	Positive regulation of biological process	63.64	4.550 *e* − 46
12	Positive regulation of defense response	24.43	4.898 *e* − 46
